# A retrospective study in adult patients with septic shock and multiple organ failure demonstrated improved 28-day survival with adjunct TPE compared to standard care alone: true effect or mediated by a negative fluid balance achieved by RRT?

**DOI:** 10.1186/s13054-020-03315-5

**Published:** 2020-10-08

**Authors:** Patrick M. Honore, Leonel Barreto Gutierrez, Luc Kugener, Sebastien Redant, Rachid Attou, Andrea Gallerani, David De Bels

**Affiliations:** ICU Department, Centre Hospitalier Universitaire Brugmann-Brugmann University Hospital, Place Van Gehuchtenplein, 4, 1020 Brussels, Belgium

We read with great interest the recent article by Keith et al. who concluded that their retrospective, observational study in adult patients with septic shock and multiple organ failure demonstrated improved 28-day survival with adjunct therapeutic plasma exchange (TPE) compared to standard care alone [1]. The 28-day mortality rate was 40% in the TPE group (TPE+) versus 65% in the standard care group (TPE−) [1]. We would like to make some comments. The authors reported that the patients who received adjunct TPE had a more favorable fluid balance at 48 h [1]. TPE is not able to induce a negative fluid balance. Patients undergoing adjunct TPE required initiation of renal replacement therapy (RRT) in 67.6% of cases, compared to 51.4% in those receiving standard of care alone [1]. The mortality associated with the new need for RRT was 48% in those receiving TPE compared to 79% in those receiving standard of care alone [1]. Almost 70% of the TPE+ patients required RRT versus only 50% of the patients in the standard of care group [1]. One of the most impressive results seen was the greater relative reduction in mortality among patients receiving TPE who had a primary sepsis diagnosis of pneumonia (pneumonia 11/23 TPE+ [mortality 47.8%] vs 15/17 TPE− [mortality 88.2%]), a situation where a negative fluid balance is so crucial [1]. Knowing that RRT is a very powerful tool to generate a negative fluid balance [2, 3], it is possible that the benefit in mortality could be linked to improved attainment of negative fluid balance in patients on RRT (70% of the TPE group) [1, 4, 5]. Naturally, this is only a hypothesis and cannot be confirmed with the available data. Fluid balance data presented in the study suggest that there were significant differences at baseline that were not matched in the TPE+ and TPE− groups [1]. Changes in fluid management over time, including the use of diuretics and cumulative duration of RRT, were not reported [1]. Individualized treatment occurred in both groups based on physician preferences (e.g., adjunct steroids, ascorbic acid, thiamine), and this was also probably the case for RRT (70% TPE+ vs 50% TPE−) [1]. It appears that the “standard of care” varied considerably in the study. In conclusion, we wonder if the observed difference in mortality was a result of negative fluid balance due to RRT.

## Data Availability

Not applicable.

## Abbreviations

TPE: Therapeutic plasma exchange
TPE+: Patient cohort receiving therapeutic plasma exchange
TPE−: Patient cohort receiving “standard of care”
RRT: Renal replacement therapy
